# The Ambiguous Roles of Extracellular Vesicles in HIV Replication and Pathogenesis

**DOI:** 10.3389/fmicb.2018.02411

**Published:** 2018-10-10

**Authors:** Marcos V. S. Dias, Cristina S. Costa, Luis L. P. daSilva

**Affiliations:** ^1^Center for Virus Research, Ribeirão Preto Medical School, University of São Paulo, Ribeirão Preto, Brazil; ^2^Department of Cell and Molecular Biology, Ribeirão Preto Medical School, University of São Paulo, Ribeirão Preto, Brazil

**Keywords:** HIV, extracellular vesicles, exosomes, microvesicles, HIV therapy, AIDS

## Abstract

Cells from all kingdoms of life can release membrane-enclosed vesicles to the extracellular milieu. These extracellular vesicles (EVs) may function as mediators of intercellular communication, allowing the transfer of biologically active molecules between cells and organisms. It has become clear that HIV particles and certain types of EVs, such as exosomes, share many similarities regarding morphology, composition, and biogenesis. This review presents a summary of the literature describing the intricate relationship between HIV and EVs biogenesis. Also, we discuss the latest progress toward understanding the mechanisms by which EVs influence HIV pathogenesis, as well as, how HIV modulates EVs composition in infected cells to facilitate viral spread.

## Definition, Origin/Biogenesis of Evs

Extracellular vesicles (EVs) are heterogeneous membrane-enclosed structures released by cells in an evolutionarily preserved fashion. Over the past decade, EVs are attracting considerable interest in the scientific community due to their ability to mediate cellular exchange of nucleic acids, proteins and lipids, thereby affecting a variety of physiological and pathological processes in recipient and/or parental cells.

**FIGURE 1:**
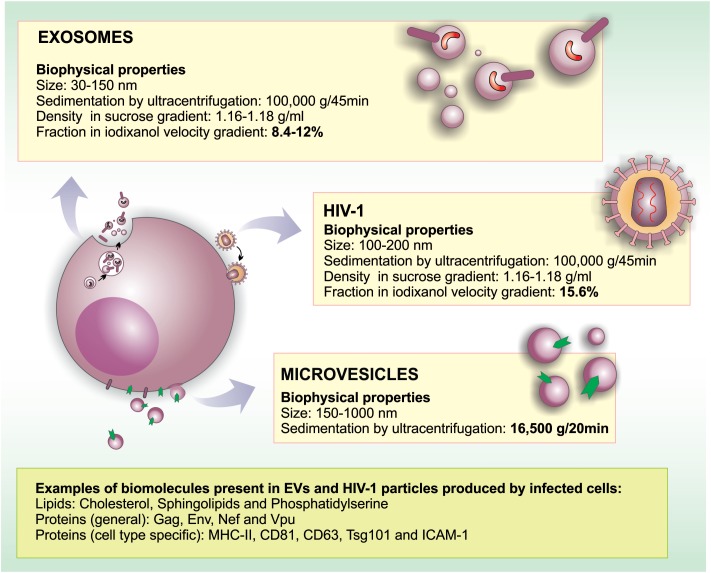
Similarities and differences in morphogenesis, biochemical and biophysical properties between EVs and HIV-1. HIV-1 particles range between 100 and 200 nm in diameter, overlapping with the size range of microvesicles (150–1,000 nm) and exosomes (30–150 nm). Regarding biochemical properties, HIV-1 and EVs are surrounded by a lipid bilayer enriched in lipid rafts microdomains. In HIV infected cells, viral particles, and EVs may contain common proteins derived from the PM (such as raft-associated proteins, MHC-II, tetraspanins, depending on the producer cell type) and ESCRT proteins. Moreover, EVs may incorporate viral proteins when formed in infected cells (Nef, Vpu, Gag, Env). Importantly, proteins such as CD45 and acetylcholinesterase (AChE) were reported not to be incorporated into HIV-1 membranes in some cell types. Similar to HIV-1 that packages its RNA genome into particles, EVs may also carry host and viral RNA molecules that can be biologically active in recipient cells.

The descriptions of membrane-enclosed structures found in the extracellular milieu started in the late 1960s; initially, this kind of cell particle release was described as a form of eliminating unneeded cellular elements during the maturation of reticulocytes (Johnstone et al., 1987). Since then, many classes of EVs have been described, and it became clear that their origin, composition, and size are well diverse. Not surprisingly, different names have been used in the literature for these vesicles (reviewed by van der Pol et al., 2016). Although the nomenclature is still a matter of debate (Colombo et al., 2014; van Niel et al., 2018), the term microvesicle generally refers to vesicles that bud directly from the plasma membrane (PM) with a diameter ranging between 150 and 1,000-nm. On the other hand, the term exosome refers to smaller (30–150 nm) vesicles derived from the endosomal system that are released from cells as a result of multivesicular endosomes/bodies (MVBs) fusion with the PM (**Figure 1**). Other vesicular structures such as apoptotic bodies, exosome-like vesicles and membrane particles are also collectively referred as EVs (reviewed by Colombo et al., 2014; van Niel et al., 2018).

Exosomes are the most well-studied class of EVs. They originate as intraluminal vesicles (ILVs) during the process of early endosome maturation, which gives rise to MVBs. MVBs typically vary from 250 to 1,000 nm in diameter and contain multiple ILVs ranging from 30 to 100 nm in diameter. These endosomal compartments may fuse with lysosomes, for ILV degradation, or with the PM releasing these ILVs into the extracellular milieu as exosomes (reviewed by Colombo et al., 2014; van Niel et al., 2018).

Distinct molecular mechanisms were proposed regarding ILVs/exosomes biogenesis. Undeniably, the best-characterized is accomplished by components of the Endosomal Sorting Complex Required for Transport (ESCRT) machinery. This evolutionarily conserved protein machinery comprises four multiprotein complexes: ESCRT-0, -I, -II, and -III, and accessory proteins (e.g., Alix and VPS4). The ESCRT-0, -I, and -II recognize and cluster monoubiquitinated transmembrane proteins at the endosomal membrane, whereas the ESCRT-III facilitates membrane invagination and scission of ILVs (reviewed by Raiborg and Stenmark, 2009). In fact, ESCRT proteins were identified in proteomic profiling data from distinct types of EVs (Kim et al., 2013), which corroborates the participation of this machinery in EVs biogenesis.

In contrast, exosomes may also be produced in an ESCRT-independent manner, since cells simultaneously depleted of key subunits of the four ESCRTs continued to form MVBs featuring ILVs (Stuffers et al., 2009). The ESCRT-independent biogenesis of exosomes may involve tetraspanin proteins and certain lipid molecules, such as ceramide. Interestingly, decreased levels of exosomes release were found in oligodendroglial cell line upon inhibition of neutral sphingomyelinases (enzymes that hydrolyze sphingomyelin to ceramide) (Trajkovic et al., 2008). In this work, the authors proposed that ILV biogenesis could be triggered by the formation of ceramide microdomains in areas of the endosome membrane containing high concentrations of sphingolipids, which would coalescence into larger ceramide-rich membrane areas prone to budding. Similar results were described using other cell lines (Chairoungdua et al., 2010; Dreux et al., 2012; Hoshino et al., 2013), demonstrating that the formation of ceramide is essential for exosome biogenesis.

Tetraspanins, a superfamily of proteins comprising 33 members in mammals, have four transmembrane domains and are known to form tetraspanin-enriched microdomains (TEMs) in membranes (reviewed by Yáñez-Mó et al., 2009). These proteins are enriched in MVBs and have been proposed to play a role on exosomes biogenesis (reviewed by van Niel et al., 2018). Mechanistically, these proteins form clusters with themselves and with other transmembrane and cytosolic proteins at membranes, acting on the formation of microdomains in a cone-like structure with an intramembrane cavity (reviewed by Charrin et al., 2014). Finally, these clusters are able to induce inward budding of the microdomain in which they are enriched (Zimmerman et al., 2016).

The mechanisms by which secretory MVBs are mobilized to the cell periphery, dock, and fuse to the PM are incompletely understood. Currently, it is known that the trafficking of MVBs to the PM involves both the actin and the microtubule cytoskeleton (Mittelbrunn et al., 2011). Moreover, Rab GTPases were shown to play a role in exosome release. Inhibition of Rab11 function in the erythroleukemia cell line K562 decreased the release of exosomes containing transferrin receptor and HSC70 (Savina et al., 2002). At least two independent studies demonstrated that Rab35 is necessary for the release of exosomes-containing myelin poteolipid protein (PLP) by a murine oligodendroglial cell line (Hsu et al., 2010) and by primary oligodendrocytes (Frühbeis et al., 2013). Additionally, an RNAi-based screening targeting the Rab GTPase family in HeLa cells, identified that knockdown of either Rab27A or Rab27B significantly decrease the amounts of CD63-containing EVs released into the extracellular milieu (Ostrowski et al., 2010). The authors also demonstrated that Rab27B regulates MVB transport toward the PM, and that both Rab27 isoforms paly a role on the docking of MVBs to the PM to promote membrane fusion, thereby increasing exosome release. Rab11 and Rab35 are mainly associated to recycling and early endosomes, respectively, whereas Rab27A and Rab27B associate with late endosomes. It has been proposed that the nature of the endosomal compartment from which exosomes originate may explain these apparently discrepant observations (reviewed by Colombo et al., 2014). In this model, Rab27A-B would act in late endosomes/MVB and mediate the release of exosomes enriched in late endosomal proteins (such as CD63, ALIX, and TSG101). Conversely, Rab35 or Rab11 would mediate the fusion of early or recycling endosomes with the PM, and the release of exosomes containing flotillin and other cell-specific proteins (such as Wnt-associated, PLP, and TfR) (reviewed by Colombo et al., 2014).

Finally, several lines of evidence indicate a close relationship between the autophagy pathway and exosome biogenesis and secretion. Autophagy stimulation can inhibit exosomal release, whereas the inhibition of autophagy seems to enhance exosomal release (reviewed by Baixauli et al., 2014). Prion protein (PRNP) is a glycoprotein anchored to the cell membrane that modulates autophagy to control exosome secretion. Specifically, PRNP appears to control the partition of caveolin-1 between lipid raft domains at the PM membrane and the cytoplasm, where caveolin-1 can function to impair the ATG12–ATG5 complex formation and thus inhibit autophagy progression. Under this condition, MVBs can fuse with the PM to release exosomes. In starvation, or absence of PRNP at the PM, caveolin-1 internalization is inhibited, and autophagy is stimulated. As a consequence, autophagosomes fuse with MVBs, and ultimately to lysosomes, thus sequestering the cellular sources of exosomes reducing the rate of their release to the extracellular milieu (Dias et al., 2016).

Because there is substantial overlap in composition and morphological features among the different types of EVs, being virtually impossible to precisely distinguish exosomes from microvesicles and other EVs in a given sample of culture media or biological fluid, we chose to adopt the general term EV, when referring to these membranous structures throughout this review.

## Evs in Viral Infections

EVs are present in most biological fluids, such as synovial fluid, breast milk, blood, urine, and saliva. Several studies have shown the facilitator effect of EVs in immune responses, and their antigen-presenting activity has also been reported (Shenoda and Ajit, 2016). EVs also play roles in other pathological conditions, such as in inflammatory diseases, via the release of cytokines (Bretz et al., 2013), and in tumor progression, facilitating the spreading of cancer cells and metastasis (Costa-Silva et al., 2015; reviewed by Martins et al., 2013).

Although many studies have assessed the roles and functions of EVs in modulating the immune system, especially in anti-tumor immunity, limited data are available that illuminate the biology of these vesicles in infectious diseases. In the case of viruses, for instance, several lines of evidence indicate that infected cells release EVs and that these EVs can be used to spread viruses. Coxsackievirus B1, a member of the *Picornaviridae* family, is able to depolymerize the host actin cytoskeleton during infection, and this disruption leads to the release of EVs and enables virus spread to secondary sites to perpetuate infection (Inal and Jorfi, 2013).

The Epstein–Barr virus (EBV), a member of the Herpesviridae family that can cause tumors in humans, induces the transfer of a viral oncoprotein, LMP-1, and virus-encoded miRNAs through EVs. Interestingly, these vesicles can manipulate the growth of neighboring cells by activating ERK and AKT signaling pathways (Meckes et al., 2010). The characterization of EVs released by EBV-infected cells, showed the presence of mRNAs coding for the latent phase viral proteins LMP1, LMP2, EBNA1, and EBNA2. These viral elements may act as signaling effector molecules and transcription factors in virus-driven cellular transformation (Canitano et al., 2013), corroborating evidence that mRNAs carried by EVs can be fully functional in target cells (Skog et al., 2008).

Extracellular vesicles were also described as carriers for *Flavivirus* transmission from arthropod to human cells (Zhou et al., 2018). *Ixodes scapularis* tick cells infected by Langat virus (LGTV), a member of the Flaviviridae family, release EVs that contain LGTV replicative virus RNA, structural (E), and non-structural (NS1) proteins. In fact, these EVs were capable of infecting human keratinocytes and human vascular endothelial cells. This study also demonstrates that LGTV can use EVs for dissemination within the vertebrate host; EVs derived from infected-brain-microvascular endothelial cells targeted neuronal cells and could disseminate infection in the central nervous system (CNS) (Zhou et al., 2018).

In contrast, EVs were shown to play an ambiguous role in certain types of viral infections, benefiting either the host or the virus. Hepatitis C virus (HCV) has evolved a strategy that prevents type 1 interferon (IFN) induction by infected hepatocytes (Liang et al., 2008). However, HCV-infected hepatocytes can selectively incorporate immunostimulatory viral RNA within EVs which then deliver these molecules to neighboring plasmacytoid dendritic cells (DCs), inducing strong interferon production that contributes to the antiviral response. This viral RNA transfer mediated by EVs is dependent on the ESCRT machinery. Thus, sequestration and release of viral RNA within EVs may aid the virus to evade pathogen-sensing mechanisms in infected cells, but also serve as host strategy to induce an unrestrained innate response in non-infected by-stander cells (Dreux et al., 2012).

## Shared Mechanisms and Crosstalk Between Evs and Hiv-1

The human immunodeficiency virus type 1 (HIV-1) belongs to the genus *Lentivirus* within the family Retroviridae, and Orthoretrovirinae subfamily (reviewed by Freed and Martin, 2013). HIV-1 debilitates the host immune system by infecting and destroying T cells and macrophages that express CD4 receptor and either the CCR5 or the CXCR4 co-receptors, leading to immunodeficiency at later stages of disease (reviewed by Chun and Fauci, 2012). Although the highly active antiretroviral therapy currently available, made it possible to control HIV-1 infection, challenges still exist due to patient’s divergent response to HIV infection and therapies, and because the eradication of latent virus reservoirs is still elusive. Therefore, it is critical to fully understand the biology of HIV-1 and its interaction with cells of the host immune system to unravel previously unexplored aspects of this virus.

Extracellular vesicles have been shown to play important roles in HIV-1 infection. In fact, EVs (especially exosomes) and HIV-1 particles share some important aspects regarding their biogenesis, biophysical/molecular properties and cellular uptake mechanisms. Among the most prominent similarities between HIV-1 and exosomes are that both particles are surrounded by a phospholipid bilayer, and their sizes range between 100 and 200 nm in diameter. This morphological resemblance makes the precise separation between HIV-1 and exosomes technically challenging, as discussed latter in the review. Nevertheless, the main differences and similarities between EVs and HIV-1 are discussed below and are summarized in **Figure 1**.

### Biophysical and Molecular Properties

Regarding molecular properties, both EVs and HIV-1 have significantly higher levels of cholesterol and glycosphingolipids than the PM (Aloia et al., 1993; Wubbolts et al., 2003), where these lipids are enriched in detergent resistant membrane, or rafts, domains. The regionalization of these lipids alters membrane structure and may be involved in the generation of membrane buds and even in membrane fission (reviewed by Huttner and Zimmerberg, 2001), characteristics that are essential to the formation of exosomes and HIV-1 particles.

A variety of typical raft-associated proteins could be detected in EVs isolated from different cell types (de Gassart et al., 2012). In particular, major histocompatibility complex (MHC) class II molecules are concentrated in EVs released by antigen presenting cells (Gauvreau et al., 2009). HLA class II is one of the most prominent cell surface proteins incorporated in HIV-1 particles during budding in cell culture (Poon et al., 2000; Ott, 2008) or *in vivo* (Saarloos et al., 1997). As mentioned before, exosomes and other EVs are highly enriched in tetraspanins. These proteins act primarily as a scaffold that laterally organizes membrane-based cellular functions (reviewed by Charrin et al., 2014). Among tetraspanins, CD63 and CD81 are classical exosome markers, whereas CD9 is also found in larger vesicles and thus not considered a bona fide marker of MVB-derived vesicles. Therefore, although tetraspanins may not be used as markers for a specific type of EV, this class of proteins may provide powerful approaches on determining EV-enrichment (reviewed by Andreu and Yáñez-Mó, 2014).

Similar to EVs, HIV-1 particles may incorporate tetraspanins such as CD9, CD63, CD81, and CD82 during their cell-exit process in epithelial cells (Grigorov et al., 2006), T lymphocytes (Jolly and Sattentau, 2007), macrophages (Deneka et al., 2007; Welsch et al., 2007) and DCs (Garcia et al., 2008). Analyses of lipid rafts from the cell surface of macrophages, as well as, from EVs and HIV-1 particles originated from these cells, revealed a robust correlation between their protein profiles. These results further indicated that typical EV markers, such as CD63, CD81, and MHC II, can also be incorporated in HIV-1 particles (Nguyen et al., 2003).

Tetraspanins are thought to regulate several steps of HIV-1 transmission. For instance, they can act as organizers of fusion platforms, allowing access of viral fusogens to PM microsegments and also by recruiting cellular factors that promote lipid bilayer curvature during virus budding and release (Singethan et al., 2008). Similarly, tetraspanins could also be involved on the bind and uptake of specific EVs to recipient cells. For example, EVs derived from a metastatic pancreatic adenocarcinoma cell line, enriched in tetraspanins CD151 and Tspan8, were shown to preferentially target lung and lymph node stroma cells, suggesting that tetraspanins at the EV surface may define structures for binding and fusion with target cells (Rana et al., 2012).

Similar to the other members of retrovirus family, HIV-1 packages its RNA genome into viral particles, which is crucial for viral replication. This full-length HIV RNA provides a template for viral genome replication and also for viral protein production. Similarly, RNA molecules (mRNA and microRNA) are packaged into EVs and were shown to be bioactive in target cells (Skog et al., 2008).

### Mechanisms of Biogenesis

As mentioned before, the ESCRT-dependent pathway is the best-characterized mechanism underlying EV biogenesis. Although ESCRTs are generally associated with the inward budding of vesicles to the lumen of endosomes, in T cells and macrophages, ESCRT proteins have been found in both endosomal compartments and the PM (Welsch et al., 2006). In fact, ESCRT proteins are required for efficient budding of HIV-1 particles from CD4+ T lymphocytes, which occurs at the PM (reviewed by Freed, 2015). To promote viral particle release, the so-called late domains, present in p6 domain of the Gag precursor protein (Pr55Gag), bind o specific ESCRT machinery components mediating their recruitment to viral budding sites. Specifically, the primary PTAP-type late domain of HIV-1 recruits ESCRT-I by binding to Tsg101 (tumor susceptibility gene 101), and an auxiliary LYPXnL (where X represents any amino acid)-type late domain recruits the ESCRT-accessory protein Alix (reviewed by Votteler and Sundquist, 2013; Freed, 2015). Interestingly, HIV-1 budding may not fully recapitulate ILV formation because only a subset of the human ESCRT-III subunits, in particular CHMP2 and CHMP4, seems to be required for viral budding (Morita et al., 2011).

The possibility that HIV-1 may be able to hijack the exosome machinery led to the Trojan exosome hypothesis (Gould et al., 2003) and explained how HIV-1 could be capable of transferring itself between cells, even in the absence of envelope glycoproteins, and scape recognition by the immune system. Interestingly, specific cell types, such as Jurkat T cells, display discrete domains of the PM that are enriched in exosomal and endosomal proteins. These PM domains possess an outward vesicle budding property that is topologically similar to the inward ILV-formation that occurs in endosomes. Therefore, in those cells, the PM may serve as a site of immediate exosome-like EV biogenesis, and HIV-1 could use these domains to form infectious particles (Booth et al., 2006).

As mentioned previously, small GTPases Rab proteins have the capacity to interfere with EV release. Rab27A and Rab27B control the release of EVs by facilitating MVB recruitment and docking to the PM (Ostrowski et al., 2010). In CD4+ T cells, Rab27A controls the trafficking of late endosomes/MVBs containing the enzyme phosphatidylinositol 4-kinase type 2 α (PI4KIIα) toward the PM. Once in the PM, this enzyme catalyzes the production of phosphatidylinositol 4-phosphate PI(4)P mediating the localized membrane-enrichment of phosphatidylinositol 4,5-bisphosphate PI(4,5)P2 molecules, which are required for the recruitment of viral polyprotein Pr55^Gag^ and HIV-1 assembly (Gerber et al., 2015). Rab27A also plays a role in PI(4,5)P2 production and viral replication in macrophages, which normally recruits Pr55^Gag^ to PM invaginations termed virus-containing compartments. Altogether, the data indicates that HIV-1 and EVs release could be controlled in similar ways.

### Cellular Uptake and Fate

Unlike HIV-1, for which viral entry specifically depends on the interaction of Env glycoprotein with CD4 receptor and cognate cellular co-receptors, limited data are available regarding the mechanisms of cellular uptake of EVs. Although mostly based on indirect evidence and *in vitro* studies, several types of interactions have been proposed between EVs and target cells (reviewed by Théry et al., 2009). EVs released by mature DCs carry intercellular adhesion molecule 1 (ICAM1) on their surface and can be captured by CD8+ DCs and activated T cells, through binding to the lymphocyte function-associated antigen 1 (LFA1) protein at the surface of those recipient cells (Segura et al., 2007). In a similar fashion, HIV-1 particles, which are derived from the PM of T cells, can display ICAM1 at its surface, which in turn bind to LFA1 expressed on the surface of target T cells, hence increasing viral infectivity (Tardif and Tremblay, 2005).

Several lines of evidence suggest that fusion-independent pathways enable cell entry of both HIV-1 and EVs. Regarding EVs uptake, many endocytic modalities have been shown to be involved, such as macropinocytosis (Fitzner et al., 2011), phagocytosis (Montecalvo et al., 2012) and clathrin-mediated endocytosis (CME) (Escrevente et al., 2011). In DCs, internalized EVs are delivered to endosomes and processed. Peptides derived from those EVs can then load MHC-II molecules for CD4+ and CD8+ T cells presentation, leading to their activation (Morelli et al., 2004). Curiously, endocytosis of HIV-1 particles has been demonstrated to result in productive infection in CD4+ T lymphocytes (reviewed by Permanyer et al., 2010). Moreover, the transmission of HIV-1 from DCs to primary CD4+ T cells may be accomplished via a trypsin-resistant endocytosis mechanisms that leads to a productive infection (Clotet-Codina et al., 2009).

Phosphatidylserine (PdtSer) is a membrane phospholipid, enriched in EVs, which binds to PdtSer receptors at the cell surface. The T-cell immunoglobulin domain containing molecules-1 and 4 (TIM-1 and TIM-4) are PtdSer receptors involved on the engulfment of apoptotic cells and EVs displaying PtdSer (Miyanishi et al., 2007). Interestingly, HIV-1 particles are also enriched on PtdSer and this phospholipid was described as an important cofactor for HIV-1 infection on monocytes/macrophages (Callahan et al., 2003). A role for PtdSer and TIM-4 in EV-induced cellular uptake of HIV-1 has been proposed (Sims et al., 2017), and will be discussed later in this review.

## Anti-Hiv-1 Effects of Evs

**FIGURE 2:**
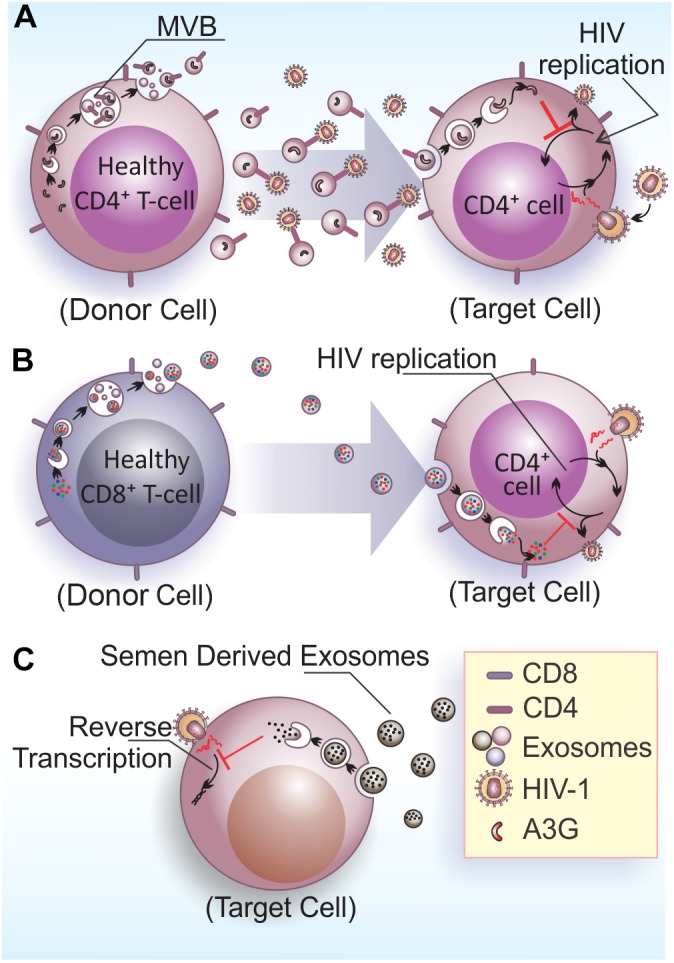
Anti-HIV-1 effects of EVs. **(A)** Exosomes released by healthy CD4+ T cells contains CD4 molecules on their surface and hinder HIV-1 infection/dissemination, possibly acting as decoys in the extracellular space. The viral factor Nef would neutralize this activity by reducing CD4 expression in EVs (De Carvalho et al., 2014). Additionally, exosomes also contain the enzyme A3G that can be internalized by infected cells and inhibit HIV-1 replication in target cells (Khatua et al., 2009). **(B)** Exosomes released from CD8+ T cells have non-cytotoxic antiretroviral activity that inhibits HIV-1 transcription in target cells (Tumne et al., 2009). **(C)** Semen derived exosomes are internalized by target cells (vaginal epithelial cells) and are able to block HIV-1 replication through the impairment of viral RNA reverse transcription (Madison et al., 2014).

In the context of viral infections, EVs play important roles in intercellular communication by signaling the presence of infectious agents and enabling antiviral responses to neighbor or long distance acceptor cells through body fluids (reviewed by Théry et al., 2009; Meckes and Raab-Traub, 2011). Some studies have pointed to a protective role of EVs against spreading of HIV-1 infection (**Figure 2**). EVs released by CD4+ T cells mediate CD4-dependent inhibition of HIV-1 infection *in vitro*. This results suggest that CD4 molecules at EVs surface can interact with HIV envelope proteins, which can hinder viral interaction with target cells, hence preventing viral infection (**Figure 2A**). Importantly, the HIV-1 accessory protein Nef reduces the amount of CD4 in EVs, diminishing their inhibitory properties (De Carvalho et al., 2014). This data suggest a role for Nef on facilitating viral spread by reducing the expression of CD4 in EVs and thus favoring HIV-1 dissemination.

APOBEC3 (A3) proteins belong to a family of cellular cytidine deaminases that have a role on restricting HIV-1 replication (reviewed by Chiu and Greene, 2008). The most prominent member of this family, APOBEC3G (A3G), has an antiretroviral function linked to its DNA-editing capacity (Miyagi et al., 2007), which can be neutralized by the HIV-1 accessory protein Vif that targets A3G for proteasomal degradation (Conticello et al., 2003). CD4+ T cells release A3G into EVs, which is able to restrict HIV-1 replication in recipient cells (**Figure 2A**) (Khatua et al., 2009). However, EVs carrying A3G may not contribute to repress HIV-1 *in vivo*, because once HIV-1 infects activated T cells, it depletes A3G from the cells and consequently from EVs, and therefore only a few EVs carrying A3G are likely to be released.

Non-cytotoxic suppression of HIV-1 replication in CD4+ T cells is an antiviral mechanism displayed by CD8+ T cells that was described decades ago (Blackbourn et al., 1996). Studies aiming to elucidate the effector molecule(s) mediating this anti-retroviral mechanism excluded CD8+ cell-secreted cytokines, chemokines, or inflammatory molecules and attributed this activity to soluble protein factors generically termed as CD8-derived antiviral factor (CAF) (Brinchmann et al., 1990; Mackewicz et al., 1994; Levy, 2003). Interestingly, CAF was later linked to EVs secreted by CD8+ T cells. Specifically, it was shown that the HIV-1 replication suppressive activity of CD8+ T cells is mediated by a protein moiety displayed at the EVs external surface and also independent of EV internalization (**Figure 2B**) (Tumne et al., 2009).

Additional insights into the possible anti-HIV effect mediated by EVs came from studies with cellular components from the blood–brain barrier (BBB), which provide physical and immunological protection against viruses that may enter the central nervous system. Strikingly, human brain microvascular endothelial cells (HBMECs), the most numerous cells of the BBB, are non-permissible to HIV-1 and can transfer anti-HIV-1 protection to permissible cells such as macrophages. This transference occurs through HBMEC-derived EVs loaded with antiviral factors including products of key IFN-stimulated genes (ISGs; ISG15, ISG56, and Mx2), both at mRNA and protein level, contributing to the set of defenses that block virus entrance into the CNS (Sun et al., 2016).

In this context, it is noteworthy that EVs are present in a wide range of human body fluids, and recent reports showed that some of those EVs can inhibit HIV-1 infection. EVs isolated from human breast milk of healthy donors were able to inhibit HIV-1 infection in monocyte-derived dendritic cells (MDDCs), acting as a protective factor against vertical virus transmission. The inhibition was likely due to the binding of EVs to the DC-SIGN receptor, which can compete with the virus and hinder MDDC-mediated viral transfer to CD4+ T cells (Näslund et al., 2014). It was suggested that the protective effect of EVs might be transferred to infants via breastfeeding as part of a passive antiviral immunity (Näslund et al., 2014).

Similar to breast milk, semen-derived EVs have antiviral effects on HIV-1 infection. EVs isolated from the semen of healthy men were internalized by recipient cells, and upon internalization, led to a blockage on HIV-1 replication through impairment of viral RNA reverse transcription (**Figure 2C**) (Madison et al., 2014). Semen EVs were also capable of blocking HIV-1 dissemination from vaginal epithelial cells to monocytic and lymphocytic cell lineages, and also to peripheral blood leukocytes. In fact, studies using cell culture and an *in vivo* LP-BM5 murine AIDS model, showed that vaginal cells were able to internalize human semen EVs that contained functional viral mRNA (Madison et al., 2015). In contrast, vaginal fluids also contains EVs that decrease HIV-1 transmission in the Jurkat cell line, blocking a post-entry step of virus infection, most likely reverse transcription (Smith and Daniel, 2016). The studies that described the anti-viral effects of breast milk and semen-derived EVs also found that blood-derived EVs were not able to restrict HIV-1 transmission (Madison et al., 2014; Näslund et al., 2014), demonstrating that antiretroviral EVs exist in some, but not all, bodily fluids from healthy individuals. Therefore, EVs derived from bodily fluids that naturally display anti-HIV-1 activity are attractive for the development of new antiretroviral therapies.

## Enhancement of Hiv-1 Infection and Pathogenesis Mediated by Evs

Although EVs derived from uninfected cells may exert protective effects against HIV-1, the virus has its own mechanisms to subvert the endomembrane system. This subversion may not only lead to enhancement of viral biogenesis itself, but also to EVs biogenesis changes. These modifications may involve alterations in cargo composition, frequency of EVs release and targeting, which may contribute to viral immune evasion and increased pathogenesis.

**FIGURE 3:**
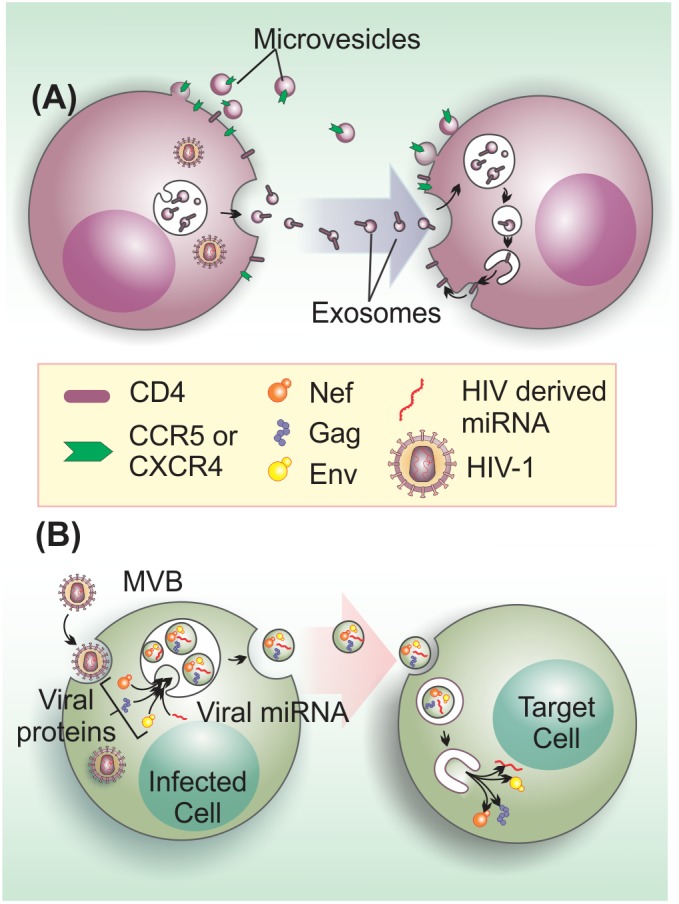
Extracellular vesicles on the enhancement of HIV-1 infection and pathogenesis. **(A)** EVs from HIV-1 infected cells transfer HIV-1 receptors to null cells to spread infection. EVs transfer the HIV-1 co-receptors CXCR4 and CCR5 and may be also involved in CD4 transfer (Mack et al., 2000; Esser et al., 2001; Rozmyslowicz et al., 2003). **(B)** HIV-1 components, such as viral proteins (Gag, Env, and Nef) and microRNAs may be transferred to target cells to enhance infection through the activation of quiescent CD4+ T cells (Arenaccio et al., 2014), apoptosis induction in bystander CD4+ T cells (Lenassi et al., 2010), or other unknown effects.

Initial evidence that EVs could enhance HIV-1 infection came from findings showing that EVs could mediate cellular transfer of co-receptors involved in viral cell entry. For instance, the chemokine receptor, CCR5, can be released via EVs from CHO (Chinese hamster ovary) cells and PBMCs (peripheral blood mononuclear cells), and these vesicles can transfer the receptor to CCR5-deficient PBMCs and endothelial cells (Mack et al., 2000). In a similar manner, platelet and megakaryocyte-derived EVs can transfer CXCR4 receptors to the surface of CXCR4-null cells (Rozmyslowicz et al., 2003). This EV-mediated transfer of host cell surface proteins can lead to infection of tissues that do not express endogenous HIV-1 co-receptors, favoring viral dissemination (**Figure 3A**).

Extracellular vesicles can also facilitate infection via viral progeny engagement, thus camouflaging it from immune surveillance. Mature HIV-1 was observed by electron microscopy in a physical association with EVs isolated from the supernatant of infected macrophages. Interestingly, this association accelerates the processes of viral infection and dissemination. The authors reasoned that this facilitator role of EVs was probably not due to the use of an alternative/direct cell-entry pathway of the virus, since CD4-lacking cells remained HIV-1 non-infected even in the presence of EVs (Kadiu et al., 2012).

Conversely, binding of EVs to PdtSer moieties exposed on the surface of HIV-1 was suggested to play a role in EV-mediated increase of infection (Sims et al., 2017). The PdtSer receptor TIM-4 is present on the surface of EVs (Sims et al., 2014, 2017) and appears to play a crucial role in this process. This notion was supported by the fact that TIM-4 blockage with antibodies, which leads to decreased HIV-1-EV interaction, hampered EV-induced HIV-1 entry in human T lymphoblastoid and macrophage cell lines (Sims et al., 2017). Interestingly, TIM-4 was shown to interact with HIV-1 Env glycoproteins (Gu et al., 2017), suggesting that both lipid and protein components of the virus envelope are involved in TIM-4 interaction.

Previous work has shown that TIM4 act as phagocytic receptor for apoptotic cells and EVs through the recognition of surface exposed PtdSer moieties (Miyanishi et al., 2007; Feng et al., 2010). However, if this is also the internalization mechanisms involved in EVs-induced HIV-1 entry remains to be investigated. In fact, a number of enveloped and non-enveloped viruses are able to hijack mechanisms of apoptotic clearance to facilitate host cell uptake and replication, a strategy known as apoptotic mimicry (reviewed by Birge et al., 2016). Because the surface exposure of PtdSer moieties, a hallmark of apoptosis, is a potent anti-inflammatory and immunosuppressive signal, the enrichment of EVs and/or HIV-1 particles displaying PtdSer in the serum of infected individuals potentially leads to systemic pro-viral effects beyond cell entry (reviewed by Birge et al., 2016).

Besides the role on aiding viral entry, EVs can also promote infection by transferring bioactive HIV-1-derived molecules to bystander cells (**Figure 3B**). For instance, a large proportion of EVs that are released by HIV-1-infected cells, contain the gp120 HIV-1 envelope (Env) protein. Intriguingly, the presence of these gp120+ EVs in HIV-1 preparations was shown to significantly increase viral infectivity in human lymphoid tissues (Arakelyan et al., 2017), via an yet unidentified mechanism. HIV-1 Gag is also an EV cargo molecule (Booth et al., 2006). In fact, higher-order oligomerization and PM binding capacity of Gag appears to be sufficient to direct this viral protein to EVs (Fang et al., 2007). Nevertheless, the effects of Gag in non-infected cells are currently unknown.

On the other hand, the non-structural Nef protein, a major determinant of HIV-1 pathogenicity that manipulates protein trafficking in host cells (reviewed by Pereira and daSilva, 2016), is the most well-known HIV-1 factor released within EVs (Campbell et al., 2008; Muratori et al., 2009; Lenassi et al., 2010; De Carvalho et al., 2014; McNamara et al., 2018). HEK293 cells expressing Nef-GFP secrete this fusion protein within EVs, which can be taken up by neighboring cells. Moreover, EVs containing Nef can also fuse with HIV-1 virions and deliver Nef protein to viral particles (Campbell et al., 2008). Interestingly, EVs-associated Nef is detected in the plasma of HIV-1-infected individuals at relatively high concentrations (Raymond et al., 2011), even when plasma HIV-1 RNA levels are undetectable (Ferdin et al., 2018). Interestingly, Nef stimulates its export by increasing of EV production (Lenassi et al., 2010). This finding corroborates and confirms previous work demonstrating that Nef increases the number of MVB in cells, which could contribute to the egress of viral particles (Stumptner-Cuvelette et al., 2003; Costa et al., 2006). Furthermore, EVs containing Nef can activate apoptosis of bystander CD4+ T cells, which may also contribute to T cell depletion during infection (Lenassi et al., 2010). This data agrees with previous findings showing that extracellular Nef proteins contribute to CD4+ T cell population decay, inducing apoptosis via CXCR4-receptor binding (James et al., 2004).

An additional mechanism by which EVs-associated Nef may promote HIV-1 spreading is by rendering quiescent bystander CD4+ T lymphocytes permissive to HIV-1 replication. ADAM17, a metalloprotease that promotes maturation of TNF-α, is loaded in a Nef-mediated manner into EVs released from primary CD4+ T cells. CD4+ T lymphocytes challenged with ADAM17/Nef EVs became susceptible to HIV-1 replication as a consequence of cell activation induced by TNF-α (Lee et al., 2013; Arenaccio et al., 2014; Ostalecki et al., 2016). In a similar manner, Nef can also promote viral replication in cells latently infected with HIV-1 (Arenaccio et al., 2015). These mechanisms are likely relevant *in vivo* (reviewed by Baur, 2011). In fact, the presence of EVs carrying Nef, ADAM17 and several pro-inflammatory factors in the plasma correlates with HIV-1-associated immune pathogenesis in both viremic and non-viremic chronic infection (Lee et al., 2016; Ostalecki et al., 2016), with the surprising finding that hepatocytes may represent a major source of these Nef/ADAM17 pro-inflammatory EVs *in vivo* (Lee et al., 2018).

HIV-1 infection tends to cause chronic neurologic disease in patients. HIV-1 can cross the BBB and enter the CNS early in infection, probably concomitant with initial systemic infection (reviewed by Boissé et al., 2008). In addition, HIV-1-infected immune cells can infiltrate the CNS and spread the virus, where microglia and astrocytes are susceptible to infection, and other cells such as neurons may also be affected. A few studies have described the role of EVs in neuroimmune pathogenesis. HIV-1-infected microglia releases EVs containing Nef, which, in turn, can disrupt BBB integrity and permeability by reducing the expression of the tight junction protein ZO-1. In addition, EVs-associated Nef induces an increase on Toll-like receptor-induced cytokines and chemokines levels (including IL-12, IL-8, IL-6, RANTES, and IL-17A in microglia) (Raymond et al., 2016). EVs containing Nef protein and mRNA were also detected in the plasma of patients presenting HIV-1-associated neurocognitive disorders (HANDs). Furthermore, plasma-derived EVs can also deliver Nef mRNA and induce Nef expression in a neuroblastoma cell line. This expression increased the production and secretion of Aβ peptides. It is possible to speculate that increased secretion of amyloid peptides could contribute to the cognitive impairment seen in HAND (Khan et al., 2016).

The incorporation of Nef in EVs was shown to be depend on an amino acid motif comprising residues 66–70 (VGFPV) at the N-terminus of Nef, termed the secretion modification region (SMR) (Ali et al., 2010). The molecular mechanism for Nef incorporation into EVs is not entirely understood, however, it seems to involve Nef binding to the cellular protein mortalin. Mortalin, a member of the heat shock 70-kDa protein family, associates with lipid rafts in the PM and regulates the intracellular trafficking of cell surface receptors, such as fibroblast growth factor 1 (FGF-1) (Mizukoshi et al., 1999). Nef binds to mortalin via the SMR motif, and small peptides derived from SMR can inhibit the release of Nef in EVs (Shelton et al., 2012).

In addition to HIV-1 proteins, other viral components are also found in EVs. HIV-1 co-evolved with the host by encoding its own miRNAs or RNAi suppressors that can inhibit host RNAi response (de Vries and Berkhout, 2008). In fact, there is a differential miRNA content in EVs from HIV-1-infected and uninfected cells (Roth et al., 2015). HIV-1 derived miRNAs, including vmiR88 and vmiR99, were detected in EVs isolated from HIV-1-infected macrophages and plasma from HIV-1 seropositive patients. These EVs miRNAs stimulate TLR8 signaling to promote TNFα release and may contribute to chronic immune activation in patients (Bernard et al., 2014). Additionally, the HIV-1 trans-activation response (TAR) RNA, a pre-microRNA that produces mature microRNAs, is incorporated into EVs isolated from HIV-1-infected cell culture supernatant and plasma from patients. Prior exposure to EVs from infected cells containing TAR, made uninfected target cells more susceptible to HIV-1 infection, since TAR from EVs is able to reduce the expression of Bim and Cdk9 proteins in target cells, hence inhibiting apoptosis (Narayanan et al., 2013).

## Potential for Evs in Anti-Hiv-1 Therapy

There is a growing body of evidence in the literature indicating that EVs play important roles in the communication between HIV-1-infected and healthy cells, stirring interest on the use of EVs as potential therapeutic tools. The presence of EVs in biological fluids suggests that these vesicles may be used as biomarkers for the diagnosis and prognosis of human diseases. In the case of HIV-1 infection, blood-derived EVs, carrying viral components, could be considered a promising biomarker regarding the progression of infection and could also be used to assess treatment efficacy. However, both EVs (especially exosomes) and HIV-1 viral particles are similar in size and density (**Figure 1**); thus, developing protocols to effectively separate these two entities has been challenging.

The standard method to isolate EVs is differential ultracentrifugation (Théry et al., 2006). However, EVs obtained by this method may be contaminated with HIV-1 particles, leading to unreliable results. Iodixanol velocity gradients have been used to segregate exosomes (in the low-density/upper fractions) from virus particles (in the high-density/lower fractions). Using this method, exosomes and other EVs in the plasma of HIV-1-infected individuals, seem to be efficiently separated from HIV-1 particles, even when virus loads are high (Cantin et al., 2008; Konadu et al., 2016). Importantly, proteins such as CD45 and acetylcholinesterase (AChE) seem not to be incorporated into HIV-1 membranes (Esser et al., 2001; Cantin et al., 2008) and may be used to further analyze the fractions by western blot. In fact, a recent study used magnetic nanoparticles coupled to antibodies against these proteins to deplete EVs from HIV-1 preparations (Arakelyan et al., 2017).

As mentioned previously, EVs that exhibit antiviral activity have been purified from breast milk (Näslund et al., 2014) and semen (Madison et al., 2014). These EVs display intrinsic protective properties that appear to restrain vertical and horizontal viral transmission. These isolated EVs could be useful as natural carriers of anti-HIV-1 molecules, thereby preventing viral infection. However, further studies are necessary to characterize how EVs derived from the various biological fluids correlate with the different pathological states of HIV-1 progression. An interesting recent finding is that the amount of EVs present in the extracellular milieu may also influence HIV production and infectivity. It was found that culturing HIV producer cells in EVs depleted media leads to increased HIV production and a more infectious viral progeny (Liao et al., 2017). The authors speculate that cells may respond to EVs scarcity triggering metabolic pathways that could induce viral particle production.

Since the ESCRT machinery is hijacked by HIV-1 and is also involved in EVs release, it could be an attractive target to the development of inhibitors. In fact, FGI-104, a small molecule that functions as a TSG101 inhibitor, is able to prevent HIV-1 pathogenesis (Kinch et al., 2009). Future investigation is required to determine the mechanistic basis of FGI-104 antiviral activity, the intracellular pathways that this molecule could impact, and the side effects that could be triggered by its use. The induction of autophagy during infection could also provide a mean to inhibit the biogenesis of EVs and, therefore, the intercellular transfer of viral molecules mediated by these vesicles. In fact, rapamycin, a specific MTOR inhibitor and inducer of autophagy, inhibits HIV-1 replication (Heredia et al., 2003). However, rapamycin has an immunosuppressive effect that limits its potential use in HIV-1 treatment, and other autophagy inducers should be evaluated as novel drug candidates to impair EV-mediated effects that benefit the virus.

Finally, the construction of synthetic EVs-like particles *in vivo* to package therapeutic agents for delivery could provide an exciting and revolutionary tool to fight AIDS. For example, an EV-based delivery system for antiviral molecules and/or therapeutic vaccines could represent a major improvement on drug development. Indeed, EVs are likely to have low immunogenicity when compared to liposomes or lentiviral-based delivery systems. Some studies have used EV-based therapeutics to treat disease, for example, by engineering EVs with antigens from human papillomavirus (HPV). This approach induced a cytotoxic T cell immune response, proving the feasibility and efficacy of this strategy (Di Bonito et al., 2015). Since 2013, many clinical trials have evaluated the therapeutic potential of EVs in different pathological conditions including Parkinson’s disease and cancer, but, despite their safety, no efficacy has been demonstrated (reviewed by Conlan et al., 2017).

## Conclusion

Although there is still much to be learned about the dual role of host EVs in HIV-1 pathogenesis, considerable knowledge has been gained in recent years. Notably, the data available suggest that EVs may inhibit HIV-1 infection/pathogenesis in the following ways. (I) EVs released from CD4+ T cells may bind the virus via the CD4 molecules on their surface hindering infection; (II) EVs derived from specific immune cells (e.g., CD4+ T cells and CD8+ T cells) or present in complex biological fluids (e.g., semen or breast milk) may deliver active molecules (e.g., A3G, CAF and also other unknown factors) to target HIV-1 infected cells restricting viral replication in different ways; (III) EVs present in body fluids (e.g., breast milk) may acts as a protective factor against HIV-1 transmission by competing with the virus for binding to surface adhesion proteins/receptors on target cells. Importantly, in HIV-1 infected cells, Nef and Vif proteins downregulate CD4 and A3G in EVs hampering their anti-HIV-1 properties. On the other hand, EVs can enhance HIV-1 infection by (I) Transferring of co-receptors CCR5 and CXCR4 to null cells; (II) Camouflaging the virus from immune surveillance through physical association; (III) Transferring of HIV-1 components (viral proteins and RNAs) to recipient cells, which induces further outcomes including T cell activation and apoptosis.

The functional relevance of EVs in HIV-1 infection remains incompletely characterized, and future studies will undoubtedly clarify various questions that remain opened. Major challenges for these studies are the difficulty to discriminate between EVs from different cellular origins in a given sample, as well as, to obtain HIV-1 preparations that are free of EVs using traditional ultracentrifugation protocols. Iodixanol velocity gradients based protocols are successful in achieving EV and HIV-1 separation, but these could be technically challenging and many studies cited here have not used this approach. These limitations must be considered when interpreting the data available. This is a very active field and ongoing studies on these topics will soon provide important additional information regarding the criteria for precise EVs and HIV particle separation.

## Author Contributions

All authors listed have made a substantial, direct and intellectual contribution to the work, and approved it for publication.

## Conflict of Interest Statement

The authors declare that the research was conducted in the absence of any commercial or financial relationships that could be construed as a potential conflict of interest.
